# Assessing health and social care needs of chronic patients in rural areas: Protocol for the CAMP mixed-methods observational study

**DOI:** 10.1371/journal.pone.0335196

**Published:** 2025-10-24

**Authors:** Angelo Cianciulli, Emanuela Santoro, Roberta Manente, Antonietta Pacifico, Marika Finizio, Nicole Bruno, Maria Costantino, Mario Capunzo, Giovanni Boccia

**Affiliations:** 1 Department of Medicine, Surgery and Dentistry “Scuola Medica Salernitana”, University of Salerno, Italy; 2 University Hospital “San Giovanni di Dio e Ruggi d’Aragona”, Salerno, Italy; 3 DAI Department of Health Hygiene and Evaluative Medicine, A.O.U. San Giovanni di Dio e Ruggi d’Aragona, Salerno, Italy; 4 U.O.C. Hospital and Epidemiological Hygiene, San Giovanni di Dio and Ruggi D’Aragona University Hospital, Salerno, Italy; University of Salerno, ITALY

## Abstract

Chronic diseases remain one of the most pressing public health challenges in Europe, disproportionately affecting older adults and residents of rural and underserved areas. Structural barriers to healthcare access, insufficient social support networks, and fragmented service delivery models amplify health disparities in these communities. In response, proximity-based and integrated care models have emerged as promising strategies, especially under national initiatives such as Italy’s National Recovery and Resilience Plan (PNRR). The CAMP (Chronic health Assessment and Mapping of Proximity needs) study is a cross-sectional, observational, non-interventional protocol designed to identify and characterize the unmet health and social care needs of adults living with chronic conditions in rural areas of Southern Italy. Using a mixed-methods approach, the study integrates standardized quantitative tools—the SF-36, EQ-5D, Barthel Index, and MSPSS—with semi-structured interviews to assess quality of life, functional autonomy, access barriers, and perceived social support. The study population includes adults aged ≥18 years with at least one chronic condition, recruited through general practitioners and social services. Descriptive and multivariate analyses will be used to explore associations between clinical and social variables, while thematic analysis will be applied to qualitative data. Expected outcomes include a comprehensive mapping of service gaps and resource distribution, as well as feasibility assessments for implementing community hospitals and telemedicine models. The findings will inform evidence-based territorial health planning and contribute to shaping more equitable and integrated care strategies for vulnerable populations. This protocol was preregistered on the Open Science Framework on May 26, 2025 (DOI: https://doi.org/10.17605/OSF.IO/YHD87).

## Introduction

Chronic non-communicable diseases (NCDs) are among the leading causes of morbidity, disability, and premature death worldwide [1]. Their burden is growing steadily, particularly in high-income and ageing societies such as Italy [2], where the prevalence of chronic conditions is exacerbated by sociodemographic transitions and structural inequalities. In rural and semi-rural contexts, limited availability of services, geographic isolation, and workforce shortages further amplify health disparities and complicate disease management [3,4].

Beyond the Italian context, recent European and OECD reports underline how unmet health and social care needs represent a persistent challenge across countries. The Health at a Glance: Europe 2024 report highlights that ageing, multimorbidity, and territorial disparities are straining healthcare systems, especially in rural areas with limited infrastructure [5]. Similarly, the OECD volume Does Healthcare Deliver? Living with Multiple Chronic Conditions (2025) emphasizes that people with multimorbidity experience poorer health outcomes, fragmented care pathways, and significant barriers to accessing integrated primary and community-based services [6].

Empirical studies confirm these trends. For instance, Maslyankov et al. (2024) documented that in Bulgaria a significant share of chronically ill adults reported unmet needs related to long waiting times, transportation difficulties, and affordability [7]. In Finland, Ilmarinen et al. (2024) found that, despite a well-developed welfare state, older adults and patients with chronic conditions continue to face obstacles related to costs, distance, and transport [8]. Comparative analyses across European countries have also shown that both formal and informal long-term care remain insufficient, leaving many individuals without adequate support [9].

Recurring themes include logistical barriers (distance, transport), economic constraints, fragmented services, and insufficient psychosocial support. These patterns reinforce the need for a mixed-methods approach: while quantitative tools capture the prevalence and distribution of unmet needs, qualitative inquiry allows exploration of patient perspectives, lived experiences, and expectations regarding service delivery. This dual perspective is particularly valuable for tailoring integrated and proximity-based care interventions, as called for by Italy’s National Recovery and Resilience Plan (PNRR) [10].

The CAMP (Chronic health Assessment and Mapping of Proximity needs) study was designed to respond to this gap. It focuses on adults living with chronic conditions in underserved areas of Southern Italy and aims to map their health-related quality of life, functional autonomy, perceived social support, and difficulties in accessing healthcare and social services. By combining quantitative tools with qualitative narratives, the study will generate data to inform place-based care planning, support the feasibility of proximity interventions, and guide policymakers toward more responsive and equitable chronic care strategies.

This manuscript outlines the protocol of the CAMP study, detailing its design, methodology, and expected contributions to both research and practice in territorial health policy.

## Materials and methods

### Aim, design and setting of the study

The CAMP study (Chronic health Assessment and Mapping of Proximity needs) is designed as a cross-sectional, observational, non-interventional study employing a mixed-methods approach. It will be conducted in multiple rural and semi-rural municipalities within the Campania region of Southern Italy, in collaboration with local health authorities and social services. For the purposes of this study, underserved rural/semi-rural areas are defined as municipalities with a medium/low population density (<150 inhabitants/km²), located more than a 30-minute drive from the nearest tertiary care hospital, and with below-average availability of health and social services, based on regional planning indicators. The classification also considers additional socioeconomic vulnerability factors, such as population aging and per capita income below the regional average. These operational criteria ensure transparency and replicability in the selection of recruitment areas.

The primary aim is to assess the health and social care needs of adults with chronic conditions residing in underserved areas, to inform future proximity-based care planning.

### Sample size, inclusion and exclusion criteria

The sample size was calculated assuming a 30% prevalence of patients reporting significant barriers to healthcare access, with a 5% margin of error and 95% confidence level. This prevalence was adopted as a conservative assumption, consistent with recent European evidence reporting unmet healthcare needs in the range of 25–35% among chronically ill and rural populations [5; 7,8]. Considering a potential dropout or non-response rate of 20–25%, we aim to enroll approximately 400–500 participants.

### Inclusion criteria

Age ≥ 18 years;

Clinical diagnosis of one or more chronic diseases (e.g., COPD, diabetes, heart failure, chronic kidney disease, dementia);

Registered with a general practitioner (GP) in the selected territories;

Ability to provide informed consent (directly or with caregiver assistance).

### Exclusion criteria

Current hospitalization or acute clinical instability;

Severe cognitive impairment without caregiver support, assessed on the basis of an existing clinical diagnosis documented in medical records or confirmed by the general practitioner. In doubtful cases, GP clinical judgment will be used to exclude patients who are unable to provide informed consent or actively participate in data collection;

Refusal to participate.

### Characteristics of participants and sample selection

Eligible participants will be identified through GP registries and social service records in the participating municipalities. There is no randomization or blinding, given the observational nature of the study. Recruitment will be coordinated by GPs and social workers, who will provide information sheets and collect written informed consent. We acknowledge that GP workload and the difficulty of reaching the most isolated individuals may represent practical barriers to recruitment. To mitigate these challenges, additional strategies will be implemented, including outreach activities through community organizations, collaboration with municipal health coordinators, and the use of snowball sampling when appropriate. These measures aim to maximize recruitment feasibility and ensure the inclusion of hard-to-reach populations.

### Study procedures and outcomes

This study does not include any interventions, experimental treatments, or comparison arms. It solely involves administering validated questionnaires and conducting interviews. After providing written informed consent, participants will be offered the choice to complete data collection either immediately at the GP’s office or social services site, or at a later scheduled appointment with the research team. Quantitative questionnaires and qualitative interviews may be administered during the same session or, when more convenient for the participant, in separate encounters within a two-week period. While the preferred mode is face-to-face, flexibility is ensured through the availability of telephone or videoconference options, particularly for participants residing in remote areas or with mobility limitations. These procedures aim to maximize feasibility, inclusiveness, and adherence across diverse rural settings.

**Quantitative data** will be gathered using the following validated instruments:

SF-36: to assess health-related quality of life [11].

EQ-5D: to measure health status and utility [12].

Barthel Index: to evaluate functional autonomy in activities of daily living [13].

MSPSS: to assess perceived social support [14].

**Qualitative data** will be collected through semi-structured interviews focused on patients’ experiences with care access, perceived needs, and expectations [15]. For the qualitative component, a purposive subsample of approximately 40–50 participants will be selected to ensure diversity in age, sex, type of chronic condition, and degree of geographic isolation. Interviews will be conducted until thematic saturation is reached, defined as the point at which no new codes or themes emerge from successive interviews. The semi-structured interview guide will include broad thematic areas such as: (i) experiences and barriers in accessing healthcare and social services; (ii) perceptions of care coordination and integration; (iii) psychosocial and family support needs; (iv) expectations and suggestions for proximity-based care models (e.g., community hospitals, telemedicine). The final wording of questions may be refined following piloting, but these domains directly reflect the study objectives and gaps identified in the literature on unmet needs among rural populations.

The primary and secondary outcomes of the study are summarized in Table 1.

**Table 1:** Primary and secondary outcomes of the study.

Type	Outcome	Measurement tool/ Source
**Primary**	Unmet health and social care needs	Semi-structured interviews; survey questionnaire
	Health-related quality of life	SF-36, EQ-5D
	Functional autonomy	Barthel Index
	Perceived social support	MSPSS
**Secondary**	Access barriers	Survey items; qualitative narratives
	Service gaps mapping	GP/social services reports; patient responses
	Feasibility of proximity-based care	Survey items; qualitative interviews

### Data management

All data will be anonymized and entered into a secure electronic database with controlled access, compliant with EU GDPR regulations. Data will be stored for at least 5 years following study completion.

### Safety considerations

No interventions or experimental treatments will be performed. Participants will only complete questionnaires and interviews, ensuring minimal risk.

### Statistical analysis

Descriptive statistics (means, SDs, frequencies) will summarize participant characteristics. Bivariate analyses (t-tests, ANOVA, chi-square) will explore associations among clinical and social variables. Multivariable logistic and linear regression models will identify predictors of poor quality of life, reduced autonomy, and limited access to care. Qualitative data from interviews will be analyzed using thematic analysis with independent coders to ensure triangulation. The study will adopt a sequential explanatory mixed-methods design. Quantitative analyses will first provide prevalence estimates and associations between clinical, functional, and social variables. Subsequently, qualitative interviews will be used to contextualize and deepen the understanding of these findings. Integration will occur at the interpretation stage through triangulation, whereby themes emerging from the qualitative analysis will be compared and contrasted with quantitative results to identify convergences, divergences, and complementarities. This approach ensures that the qualitative strand enriches the quantitative findings, providing a more comprehensive understanding of unmet needs in rural chronic care.

### Ethical considerations

The study was approved by the Campania 2 Ethics Committee (Approval no. 0013474−2025, 21 May 2025). All participants will provide written informed consent. The study was registered on the Open Science Framework (DOI: https://doi.org/10.17605/OSF.IO/YHD87).

### Study status and timeline

At the time of manuscript submission, participant recruitment has not yet begun. The recruitment phase is planned to start in September 2025, with an estimated end by December 2025. Data collection will continue until March 2026, and results are expected to be available by June–July 2026. No recruitment or data collection activities have been undertaken prior to registration or submission. Qualitative interviews will be conducted with a purposive subsample of approximately 40–50 participants rather than with the entire cohort, thereby reducing the workload. Should recruitment or interview scheduling take longer than anticipated, the timeline allows for a potential three-month extension (until June 2026) to ensure adequate coverage. This contingency plan is designed to safeguard feasibility without compromising data completeness or methodological rigor.

## Expected results

The CAMP study is expected to produce a comprehensive dataset describing the clinical, functional, and social characteristics of adults with chronic illnesses residing in rural and semi-rural Southern Italy. We anticipate identifying a high prevalence of unmet needs in areas such as health-related quality of life, access to services, and perceived social support. It is expected that older age, lower education level, and geographic remoteness will be significantly associated with worse outcomes on SF-36 and Barthel Index scores.

The study also anticipates that a notable proportion of participants will report difficulties in accessing health services, with long waiting times, travel distances, and limited domiciliary support emerging as key barriers. Thematic analysis of qualitative data is expected to yield recurring patterns related to care fragmentation, lack of service integration, and caregiver burden.

These findings will allow the generation of territorial maps indicating service gaps and population clusters most at risk, thus supporting the planning of localized interventions such as the implementation of community hospitals, multidisciplinary teams, and telemedicine services.

## Discussion

This study protocol outlines a robust methodological framework for assessing the unmet health and social care needs of chronically ill patients living in underserved rural areas of Southern Italy. By applying a mixed-methods approach and integrating both validated quantitative measures and qualitative narratives, the CAMP study addresses critical knowledge gaps in the design of territorial health interventions.

The expected findings may reveal not only the extent of service accessibility issues and care fragmentation but also socio-demographic patterns that affect chronic disease management outcomes. If these expectations are confirmed, they will reinforce the need for implementing locally adapted, proximity-based models of care, such as community hospitals and telehealth services. These models are aligned with national health system reforms advocated under Italy’s PNRR, which emphasizes equitable, decentralized, and multidisciplinary care delivery.

One of the key strengths of the CAMP protocol is its replicability across similar rural or semi-rural settings, both nationally and in other European contexts. The stratified sampling and use of validated instruments enhance the generalizability of findings, while the inclusion of semi-structured interviews adds contextual depth.

Nevertheless, some limitations must be acknowledged. Being a cross-sectional observational study, causal relationships cannot be inferred. Response bias may arise from self-reported instruments and voluntary participation, potentially underrepresenting more fragile subgroups. Moreover, logistical challenges in reaching the most isolated populations may affect full territorial representation.

Despite these limitations, the CAMP study has the potential to inform actionable strategies for improving chronic care delivery, resource allocation, and integration between social and health services. It also contributes methodologically by demonstrating how mixed-methods research can support evidence-based health planning in complex territorial contexts.

Results will be disseminated through peer-reviewed open access publications and presentations at national and international conferences. Any protocol amendments or interruptions will be promptly reported to the Ethics Committee and updated in the OSF registry.

## Conclusions

The CAMP study protocol provides a structured and rigorous foundation for investigating chronic care needs in rural and underserved areas. Through its integrative methodology, it will generate multidimensional evidence capable of informing localized, patient-centered health strategies. The findings are expected to support the national agenda for proximity healthcare and offer transferable insights for health systems facing similar territorial inequities.
